# Supplementary material for: Pillar arrays as tunable interfacial barriers for microphysiological systems
Source: Res Sq. 2025 Jan 16:rs.3.rs-5776581. Preprint. [Version 1] doi: 10.21203/rs.3.rs-5776581/v1 (PMC11774470; doi:10.21203/rs.3.rs-5776581/v1)
Supplement: Supplement 1 [file NIHPPrs5776581v1-supplement-1.pdf]

SUPPLEMENTARY

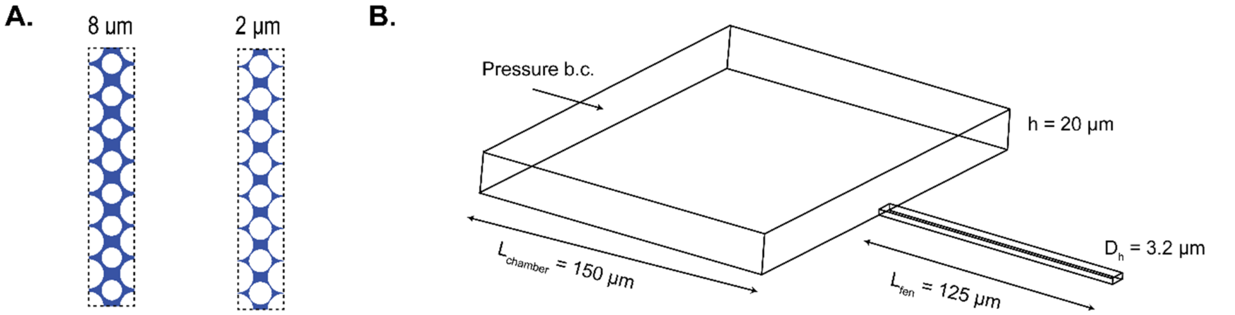

Figure S1: (A) Variation of pore size as defined by the distance between the pillars changes the porosity of the pillar-based fenestration layer. Shown here are two interfaces created in a 125 µm x 708 µm rectangle with 8 µm and 2 µm pore sizes and porosity of 31% and 19%, respectively. **(B)** A single microchannel-based fenestra finite element model was used to simulate the movement of the air-water interface across the microfluidic barrier to quantify burst pressure.

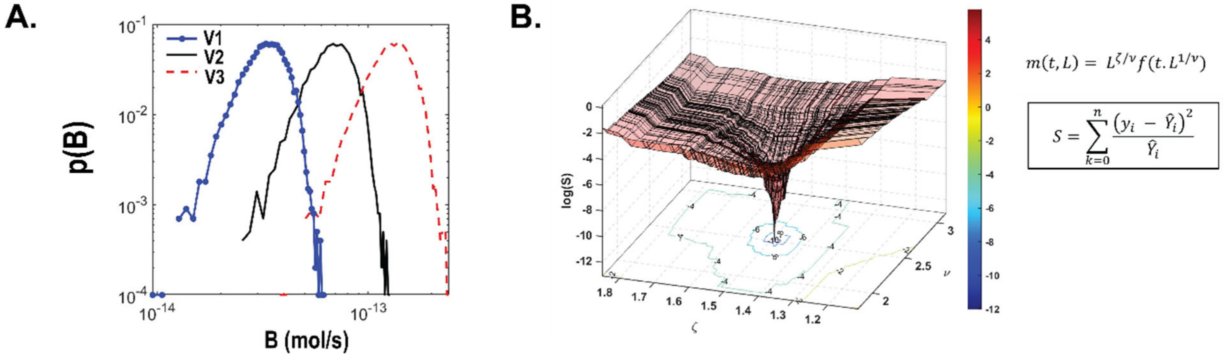

Figure S2: (A) Tissue OCR B probability distributions obtained for V1, V2, and V3 using FEM PoM method. **(B)** Differential evolution approach was used to obtain parameters for probability collapse. Show here is the estimation of parameters ν and ζ for the collapse of probability distributions of m to estimate the scaling with respect to L and t, described by a scaling function f.
